# Web-based physiotherapy for people with axial spondyloarthritis (WEBPASS) – a study protocol

**DOI:** 10.1186/s12891-016-1218-1

**Published:** 2016-08-24

**Authors:** L. Paul, E. H. Coulter, S. Cameron, M. T. McDonald, M. Brandon, D. Cook, A. McConnachie, S. Siebert

**Affiliations:** 1 School of Medicine, Dentistry and Nursing, University of Glasgow, Scotland, G12 8LL UK; 2 Rheumatology Service, NHS Greater Glasgow and Clyde, Scotland, UK; 3 National Ankylosing Spondylitis Society, 4 Albion Court, Hammersmith, London, W60QT UK; 4 Robertson Centre for Biostatistics, University of Glasgow, Boyd Orr Building, University Avenue, Glasgow, Scotland UK; 5 Institute of Infection, Immunity and Inflammation, University of Glasgow, Sir Graeme Davies Building, 120 University Place, Glasgow, G12 8TA Scotland UK

**Keywords:** Axial Spondyloarthritis, Physiotherapy, Exercise, Adherence, Internet

## Abstract

**Background:**

Evidence suggests people with axial spondyloarthritis (axial SpA) should exercise up to five times per week but lack of time, symptoms, cost and distance are barriers to regular exercise in axial SpA. Personalised exercise programmes delivered via the internet might support people with axial SpA to reach these exercise targets. The aim of this study is to investigate the effect of, and adherence to, a 12 month personalised web-based physiotherapy programme for people with axial SpA.

**Methods:**

Fifty people with axial SpA will be recruited to this prospective, interventional cohort study. Each participant will be assessed by a physiotherapist and an individualised exercise programme set up on www.webbasedphysio.com. Participants will be asked to complete their programme five times per week for 12 months. With the exception of adherence, data will be collected at baseline, 6 and 12 months.

**Discussion:**

The primary outcome measure is adherence to the exercise programme over each four week cycle (20 sessions maximum per cycle) and over the 12 months. Secondary measures include function (BASFI), disease activity (BASDAI), work impairment (WPAI:SpA), quality of life (ASQoL, EQ5D), attitude to exercise (EMI-2, EAQ), spinal mobility (BASMI), physical activity and the six minute walk test. Participants will also be interviewed to explore their adherence, or otherwise, to the intervention.

This study will determine the adherence and key clinical outcomes of a targeted web-based physiotherapy programme for axial SpA. This data will inform clinical practice and the development and implementation of similar programmes.

**Trial registration:**

ClinicalTrials.gov: NCT02666313, 20th January 2016

## Background

Ankylosing spondylitis (AS) is a chronic inflammatory arthritis associated with pain and stiffness in the spine and/or sacroiliac joints. Diagnosis of AS requires evidence of established radiographic sacroilitis which is often not evident. More recently, the term axial spondyloarthritis (axial SpA) has been proposed which encompasses radiographic disease (i.e. AS) or non-radiographic axial SpA [1]. As well as pain, reduced spinal mobility, fatigue and a reduction in physical activity, axial SpA has psychological consequences such as depression and adversely affects quality of life [2, 3].

Exercise is an effective, non-pharmacological treatment strategy for people with AS, however the optimal delivery strategy is unknown [4–7]. Whilst maximum benefit is obtained from exercising for 30 min, five times per week [8, 9], a recent consensus statement on exercise in AS recommended that adherence to regular exercise is important for self-management and therefore the exercise dose should be realistic [6]. As this patient population are increasingly diagnosed at a younger age, lack of time, symptoms such as fatigue, costs and distance to the exercise/clinical facility have been identified as barriers for people with axial SpA to regularly attend traditional physiotherapy sessions and/or to exercise regularly [10]. A Cochrane review concluded that both home and supervised exercises were beneficial for people with AS [11]. In addition, a number of studies have demonstrated that home-based exercise programmes effectively improve clinical outcomes and health-related quality of life in people with axial SpA [9, 12, 13].

In the United Kingdom (UK), 87.9 % of adults have access to the internet and have used it in the last three months [14]. The internet may therefore provide an opportunity to deliver personalised, tailored exercise programmes for people with axial SpA. Web-based interventions have the added advantage of being available 24 h a day, thereby giving flexibility to choose when and where to exercise. Web-based interventions have been investigated in osteoarthritis (OA) [15] and rheumatoid arthritis (RA) [16] where they have shown promising results in improving adherence to exercise.

We have developed a platform for delivering individualised web-based physiotherapy (www.webbasedphysio.com), the short-term effectiveness of which has been evaluated in a number of patient groups including Multiple Sclerosis [17] and Spinal Cord Injury [18]. However, whilst physiotherapy-led, personalised web-based exercise may be an effective alternative to conventional physiotherapy to support people with axial SpA to fulfil the exercise recommendations; the usage of web-delivered interventions decreases over [19]. It is therefore important to investigate the long-term adherence to, and effectiveness of, web-based exercise programmes for people with axial SpA.

The aims of this prospective, intervention, cohort study are to assess adherence to a 12 month individualised web-based physiotherapy programme for people with axial SpA, to investigate the effects of the intervention on pain, disease activity and functional activity and to determine whether there is an association between the level of adherence to the programme and these outcomes. In addition, participants’ views of the web-based intervention will be explored with special consideration of factors affecting adherence to the intervention.

## Methods

### Participants

For this prospective, intervention, cohort study, 50 participants will be recruited from the secondary-care rheumatology out-patient service of NHS Greater Glasgow and Clyde, Scotland, UK. Participants will be included if they have had a confirmed diagnosis of axial SpA for more than one year and have access to the internet at home. Participants will be excluded if they are already exercising regularly (three or more times per week), have had a joint replacement within the last six months, have any other significant comorbidities that may preclude them from taking part in a regular exercise programme or are currently participating in another clinical trial. Written informed consent will be obtained from each participant. The study has been reviewed and approved by the West of Scotland Research Ethics Committee (Ref: 15/WS/0229) and registered with ClinicalTrials.gov: NCT02666313.

### Sample size

As there are no defined criteria for determining acceptable levels of compliance, the sample size calculation has been based on best clinical judgement. Based on two thirds of patients (65 %) complying to the programme by completing an average of three exercise sessions per week in each four week period, if 50 participants are recruited to the study, then a 95 % confidence interval will have a width of ±13.2 %.

### Recruitment

Each month approximately 90 patients with axial SpA attend out-patient rheumatology and physiotherapy services in NHS Greater Glasgow and Clyde. The recruitment target of 50 participants recruited over an 8 month period will be achieved if 7 % of those attending agree to participate. Alongside recruitment from within dedicated axial SpA out-patient clinics, potential participants will be informed about the study through the University website, social media and promoted through the National Ankylosing Spondylitis Society (NASS) website, newsletters and local branches.

### Description of the web platform

**Fig. 1:**
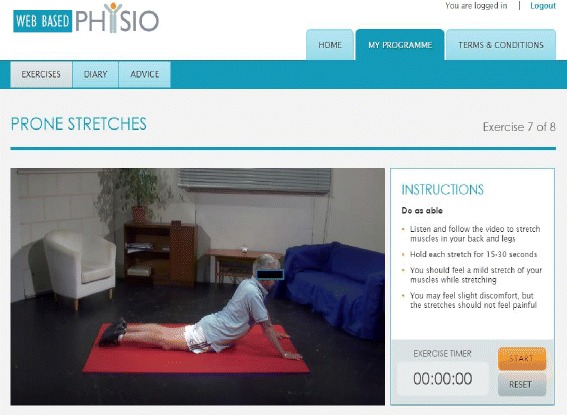
Example of user interface for exercise page on the website (www.webbasedphysio.com)

The website (www.webbasedphysio.com) consists of a home page, exercise pages, exercise diary and an axial SpA-specific advice/information section with links to relevant external websites. (The demonstration site can be viewed using the following log-in details; email axspapatient@gmail.com, password password.) Each exercise page contains a video demonstration of the exercise, a text and audio description of the exercise and a timer (Fig. 1). The website contains a catalogue of exercises with different levels of difficulty, as well as a warm up and cool down, from which the physiotherapist can create an individualised programme appropriate for each participant. The website can be easily accessed via a personal computer, tablet, smart phone or television. For this study the exercise catalogue has been expanded to include axial-SpA-specific exercises based on the *Back to Action* programme (http://nass.co.uk/back-to-action) produced by the National Ankylosing Spondylitis Society.

The website incorporates a number of behavioural change techniques known to be successful in promoting and maintaining exercise behaviour. The behavioural change techniques, operationalised within the website, have been mapped with the behavioural change techniques taxonomy proposed by Michie et al. [20]. Currently the website incorporates goals and planning, feedback and monitoring, shaping knowledge, natural consequences, comparison of behaviours, repetition, substitution and antecedents.

### Intervention

Following completion of baseline assessments (described below) participants will be reviewed by the study physiotherapist (MM), who is an experienced practitioner working with axial SpA patients. Depending on the clinical assessment, specific exercise goals will be agreed between the participant and the physiotherapist. The physiotherapist will then select appropriate exercises from the website exercise catalogue to address these goals. Participants will be provided with a unique log-in to access the website and their personalised exercise programme and axial SpA advice/information pages. Participants will be encouraged to undertake their exercise programme up to five days per week for 30 min per day [8]. Participants will complete online exercise diaries each time they complete their programme. The exercise diaries are reviewed remotely by the physiotherapist and, dependent upon progress, exercises can be progressed, added or removed from the participant’s programme. Participants will receive weekly phone calls from the physiotherapist for the first two weeks of the programme to check on progress and answer any questions. Thereafter, the online exercise diary of each participant will be reviewed every two weeks by the physiotherapist and the exercise programme remotely altered, as appropriate. Participants may also contact the physiotherapist should they feel that they require further advice or revisions to their programme.

### Study visits

**Fig. 2:**
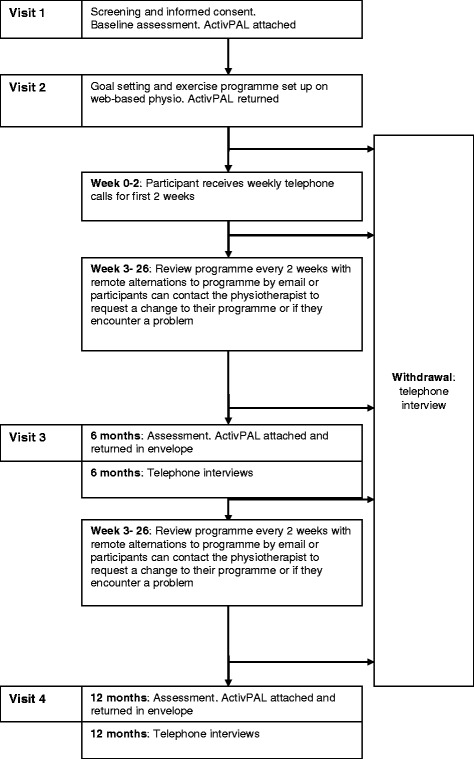
Participant journey through the study

The participant journey through the study is shown in Fig. 2. Participants will be enrolled in the study for one year and will be required to attend the physiotherapy department at their local hospital four times throughout the study.

#### Visit 1 – screening and baseline assessment (week 1)

Participants are screened to ensure they fulfil the study inclusion and exclusion criteria. Written informed consent is obtained and baseline assessments (described below) will be completed by the independent assessor (SC). As this is a cohort study and all participants receive the intervention, blinding is not relevant, however to reduce possible bias a researcher (SC) will undertake the assessments independent of the physiotherapist (MM).

#### Visit 2 – exercise goals and web-based physiotherapy programme setup (week 2)

Participants will return one week later to an appointment with the physiotherapist. Axial SpA specific exercise goals (including mobility, flexibility, and cardiovascular health) will be agreed and exercise programme devised by the physiotherapist to address these goals. Participants will then be advised on the use of the website and taken through their online exercise programme.

#### Visit 3 – midway assessment (6 months)

Outcome measures will be repeated by the assessor.

#### Visit 4 – post intervention assessment (12 months)

At the final visit the outcome measures will be repeated by the assessor.

### Outcome measures

#### Primary outcome measure

The primary outcome measure will be participant adherence to their web-based physiotherapy programme. Currently there is no consensus on the best method of measuring or reporting adherence to interventions such as a home exercise programme [21]. For the purpose of this study, adherence will be based on the number of times the participant completes their exercise diary (as recorded on the website) during each four week period, where the prescribed frequency would be 20 sessions (four weeks at five sessions per week). Each participant will complete 13 four-week periods over the course of the study. The overall compliance throughout the study will be calculated, up to a maximum of 260 exercise sessions (52 weeks at 5 times per week).

### Secondary outcome measures

#### Self-reported questionnaires

Seven self-reported questionnaires will be administered (see Table 1). The responses to the questionnaires will provide important information on disease-specific variables, as well as the participant’s perceptions of exercise.

**Table 1:** Summary of self-report questionnaires

Questionnaire	Number of items	Variable
Bath Ankylosing Spondylitis Functional Index (BASFI)	10	A validated self-report questionnaire evaluating the ability to function and cope with the activities of daily living over the past week. The questionnaire utilises 100 mm visual analogue scales ranging from no functional limitations (0 mm) to severe functional limitations (100 mm). A higher overall score indicates worse function due to more severe disease. [27]
Bath Ankylosing Spondylitis Disease Activity Index (BASDAI)	7	A validated self-report questionnaire evaluating disease activity. Visual analogue scales from none (0 mm) to very severe (100 mm) are used to assess severity of fatigue, spinal and peripheral joint pain, localised tenderness and morning stiffness over the past week. A higher overall score indicates more severe disease activity [28]
ASQoL	18	A validated self-report questionnaire addressing the physical and psychological impact of the disease; includes items relating to sleep, mood, coping, relationships, social life and activities of daily living. Scores range from 0 to 18 with higher scores reflecting poorer quality of life [29].
Work, Productivity and Activity Impairment in AS (WPAI:SpA)	6	A validated self-report, disease-specific questionnaire generating scores for absenteeism, presenteeism (reduced productivity while at work), an overall work impairment score (combining presenteeism and absenteeism) and impairment of activities outside of work during the past 7 days. Higher scores indicate more impairment [30].
EQ-5D	6	A validated self-report questionnaire measuring health status comprising two parts: (1) mobility, self-care, usual activities, pain/discomfort and anxiety/depression. Each dimension is indicated as no problems, some problems or extreme problems (2) visual analogue scale of self-rated health status from worst health imaginable (0 mm) to best health imaginable (100 mm) [31].
Exercise Attitude Questionnaire-18 (EAQ-18)	18	A self-report questionnaire evaluating how a person’s attitude affects their compliance to exercise. Items are scored reflecting three different components of attitude: affective, behavioural and cognitive. Each question is scored from 1, ‘don’t agree at all’ to 4, ‘agree very much’. A higher score indicates a positive attitude to exercise [32, 33].
Exercise Motivations Inventory – 2 (EMI-2)	51	A self-report questionnaire evaluating the extent to which a person is motivated to exercise. The components include stress management, weight management, enjoyment, appearance, personal development, ill-health avoidance and fitness. Each item is rated from 1, ‘not true at all’ to 5, ‘very true’. Items from each component are combined to obtain a mean score with higher scores representing greater motivation [34].

#### Spinal mobility

The Bath Ankylosing Spondylitis Metrology Index (BASMI) provides a clinical measurement of spinal mobility. Five different measurements of the posture and movement of the spine (tragus to wall, lumbar side flexion, lumbar flexion, cervical rotation, intermalleolar distance) will be taken by the assessor who is trained in the measurements. The resultant score out of ten indicates the participant’s limitation of spinal movement due to axial SpA [22].

#### Exercise capacity

Exercise capacity will be assessed using a six minute walk test (6MWT) which measures the total distance walked in six minutes on a hard, flat surface [23]. The participant is instructed to walk around two cones positioned 10 m apart for six minutes. They are permitted to slow down or rest when necessary and to use walking aids as required. Although not specific to axial SpA, this test is a well-recognised and validated outcome measure in a range of chronic conditions [24].

#### Physical activity

Physical activity levels will be objectively measured for one week using an activPAL activity monitor (PAL Technologies, Glasgow UK). This small, lightweight tri-axial accelerometer device is worn continuously on the front of participants’ thigh, measuring steps taken and sit-to-stand transitions, and categorises posture as either sedentary (sitting or lying), standing or walking [25]. The activPAL will be attached at each assessment using a Tegoderm waterproof dressing and participants will be asked to undertake their usual activity for seven days and then to return it to the physiotherapist (Visit 2) or return by post in a pre-paid envelope (Visit 3 and 4).

#### Telephone interviews

Semi-structured telephone interviews will be undertaken with 10 participants at six and twelve month assessment points. A sample of maximum variation based on compliance rates will be taken to select these participants. The interview will gather participants’ views of the web-based physiotherapy programme in relation to factors affecting adherence to the intervention. Issues directly related to the programme itself will be explored such as ease of use, content, any difficulties encountered and contact with the physiotherapist. Interviews will be audio recorded, transcribed and verified. Emerging themes and subthemes will be identified and agreed between two researchers (MM and LP).

In addition, participants who withdraw from the study will be asked to take part in a telephone interview to explore the reasons for withdrawal.

#### Data management/ quality assurance

All participant information and data collected will be handled according to the Data Protection Act 1998 and Good Clinical Practice. All participant data will be anonymised and any data pertaining to the participants identity will be stored in a locked filing cabinet in a locked room at the University of Glasgow. Anonymised data will be stored on a secure, password protected drive on a University server. Only the research team will have access to the data collected for the study. Upon completion of the study, the anonymised data will be labelled and stored securely in the University of Glasgow for a period of 5 years.

The proposed intervention has previously been evaluated in a multiple sclerosis patient group where no specific safety issues were found; therefore we are confident that no STOP decisions will be required. Whilst there are small, inherent health and safety issues in carrying out a home based exercise programme, as in standard clinical practice, these risks will be discussed with patients for each exercise, which is demonstrated by the physiotherapist. Additionally the physiotherapist will check that the patient can perform the exercise safely and a written and audio description of how to correctly perform each exercise is provided on the website. The study exclusion criteria will ensure that participants for whom exercise may be associated with a greater risk are not included. Any adverse events will be monitored and recorded. For serious adverse events, the chief investigator will notify the project sponsor.

A project steering committee comprising of independent clinical experts, the chief investigator, grant co-applicants and two patient representatives will oversee all aspects of the project to monitor progress and help to ensure the aims and objectives are achieved.

#### Statistical analysis

Descriptive statistics and appropriate mixed effects regression methods will be used to explore associations between baseline data, compliance and patient outcomes. The characteristics of participants who do not provide follow-up data will be compared to those who do, to assess the possible extent of non-response bias. Sensitivity of the main analyses to loss at follow-up will be assessed using multiple imputation of outcomes based on compliance and other data collected at earlier time points.

#### Dissemination

Progress and results will be disseminated at national and international conferences and a relevant peer reviewed journal. The research team will also disseminate the results to NHS staff working in rheumatology and third sector. The results of this study will also be used to inform the use and efficacy of long-term use of web-based physiotherapy and may inform its use as a clinical tool.

## Discussion

The main barriers to exercise for people with axial SpA are reportedly lack of time, fatigue, increase in symptoms, costs and distance to the exercise facility [10]. Delivering exercise programmes via a web-based platform has the potential to reduce the identified barriers, however, to the best of our knowledge, this has not been investigated in this patient cohort. The advantages of web-based physiotherapy include the convenience of the exercise programme being available 24 h a day and easy access through any portable computer, tablet, smart phone or smart television with no requirement for additional hardware. The platform may be particularly useful for those who live in remote and rural areas where attending supervised exercise sessions is associated with significant travel time, effort and costs.

This study will investigate adherence to, and the effects of, a long-term web-based exercise programme for axial SpA. To our knowledge, neither of these components has been investigated to date in people with axial SpA. It is unlikely that a simple standardised programme will be appropriate, or safe, for all patients, so the customisable nature of this programme allows for the creation of individualised programmes for each participant.

Adhering to an exercise programme requires individuals to change their lifestyle/behaviour in some way to accommodate exercise [26]. Our web-based physiotherapy platform incorporates theoretically based, behavioural change techniques to support and encourage long-term adherence to exercise [20]. Furthermore, participant interviews will explore individual factors which affect adherence and will have the potential to help guide health care professionals with strategies to improve adherence, for instance SMS/text reminders or group booster sessions [19].

There are a number of potential limitations to this study. The advantage of a prospective cohort study is that all participants receive the intervention which aids recruitment. However, by definition, there is no control group against which to compare any effects observed. In addition, people interested in exercise are more likely to enrol in the study which potentially introduces bias and affects the generalisability of the results. Conversely, people already exercising regularly will not be eligible for this study, which may affect generalisability in the other direction and mitigate the above effect. Finally, although use of mobile devices and internet access are relatively ubiquitous, only those who are able to access the internet will be recruited.

By mitigating against the previously identified barriers to exercise, web-based physiotherapy has the potential to support people with axial SpA to exercise regularly on a long-term basis at a time and venue of their choosing, thus improving clinical outcomes. Although a subsequent randomised controlled trial would be required to confirm improved efficacy for clinical outcomes, the role of exercise is already well-established in AS and axial SpA, so should this study show promising results, web-based physiotherapy may be a suitable service delivery model for people with axial SpA and to other rheumatological conditions.

## Abbreviations

6MWT: Six minute walk test
AS: Ankylosing spondylitis
Axial SpA: Axial spondyloarthritis
BASDAI: Bath Ankylosing spondylitis disease activity Index
BASFI: Bath Ankylosing spondylitis functional index
BASMI: Bath Ankylosing spondylitis metrology index
EMI-2: Exercise motivations inventory – 2
EAQ-18: Exercise attitude questionnaire-18
NASS: National Ankylosing spondylitis society
WPAI:SpA: Work, productivity and activity impairment in AS
